# Nonsense mutation in the novel *PERCC1* gene as a genetic cause of congenital diarrhea and enteropathy

**DOI:** 10.1007/s00439-022-02486-1

**Published:** 2022-09-09

**Authors:** Dina Marek-Yagel, Emily Stenke, Ben Pode-Shakked, Cara Dunne, Ellen Crushell, Anthea Bryce-Smith, Michael McDermott, Maureen J. O’Sullivan, Alvit Veber, Mansa Krishnamurthy, James M. Wells, Yair Anikster, Billy Bourke

**Affiliations:** 1 Metabolic Disease Unit, Edmond and Lily Safra Children’s Hospital, Sheba Medical Center, Tel-Hashomer, Ramat Gan, Israel; 2 Clalit Research Institute, Ramat Gan, Israel; 3 Sackler Faculty of Medicine, Tel-Aviv University, Tel-Aviv, Israel; 4 National Centre for Paediatric Gastroenterology, National Children’s Research Center, Children’s Health Ireland-Crumlin, Dublin, Ireland; 5 Division of Developmental Biology, Cincinnati Children’s Hospital Medical Center, Cincinnati, OH USA; 6 Department of Gastroenterology, St James’ Hospital, Dublin, Ireland; 7 National Centre for Inherited Metabolic Disorders, Children’s Health Ireland-Temple Street, Dublin, Ireland; 8 Department of Histopathology, Children’s Health Ireland-Crumlin, Dublin, Ireland; 9 Center for Stem Cell and Organoid Medicine (CuSTOM), Cincinnati Children’s Hospital Medical Center (CCHMC), Cincinnati, OH USA; 10 Division of Endocrinology, Cincinnati Children’s Hospital Medical Center (CCHMC), Cincinnati, OH USA; 11 The Wohl Institute for Translational Medicine, Sheba Medical Center, Tel-Hashomer, Ramat Gan, Israel; 12 School of Medicine, University College Dublin, Dublin, Ireland

## Abstract

Congenital diarrheas and enteropathies (CODEs) constitute a heterogeneous group of individually rare disorders manifesting with infantile-onset chronic diarrhea. Genomic deletions in chromosome 16, encompassing a sequence termed the ‘intestine-critical region (ICR)’, were recently identified as the cause of an autosomal recessive congenital enteropathy. The regulatory sequence within the ICR is flanked by an unannotated open reading frame termed *PERCC1*, which plays a role in enteroendocrine cell (EEC) function. We investigated two unrelated children with idiopathic congenital diarrhea requiring home parenteral nutrition attending the Irish Intestinal Failure Program. Currently 12 and 19-years old, these Irish male patients presented with watery diarrhea and hypernatremic dehydration in infancy. Probands were phenotyped by comprehensive clinical investigations, including endoscopic biopsies and serum gastrin level measurements. Following negative exome sequencing, PCR and Sanger sequencing of the entire coding region and intron boundaries of *PERCC1* were performed for each proband and their parents. In both patients, serum gastrin levels were low and failed to increase following a meal challenge. While no deletions involving the ICR were detected, targeted sequencing of the *PERCC1* gene revealed a shared homozygous c.390C > G stop gain variant. We report clinical and molecular findings in two unrelated patients harboring a shared homozygous variant in *PERCC1*, comprising the first description of a point mutation in this gene in association with CODE. That both parenteral nutrition dependent children with unexplained diarrhea at our institution harbored a *PERCC1* mutation underscores the importance of its inclusion in exome sequencing interpretation.

## Introduction

Congenital diarrheas and enteropathies (CODEs) constitute a heterogeneous group of individually rare disorders manifesting with devastating chronic diarrhea in infancy. Next Generation Sequencing (NGS) technologies have improved our understanding of the genetic architecture underlying CODEs, and accurate and timely molecular diagnoses are within reach (Thiagarajah et al. 2018; Ye et al. 2019; Esposito et al. 2021). An increasingly recognized subgroup of CODEs is caused by genetic defects affecting the enteroendocrine cells (EECs), collectively the largest endocrine organ in the body (Caralli et al. 2021; Sanchez et al. 2022). The identification of EE dysfunction as the basis of congenital diarrhea opens up the potential for novel therapeutic strategies in these patients.

Intractable Diarrhea of Infancy Syndrome (IDIS) had long been recognized as a congenital diarrheal disorder mainly affecting families of Iraqi-Jewish descent (Avery et al. 1968; Straussberg et al. 1997), however, its molecular basis remained elusive. In 2019, we identified genomic deletions in chromosome 16, encompassing a sequence which we termed the ‘intestine-critical region (ICR)’, as the cause of IDIS (Oz-Levi et al. 2019). The regulatory sequence within the ICR is flanked by an unannotated open reading frame, Proline- and Glutamate-Rich Protein with Coiled-Coil Domain 1 (*PERCC1*), which was not yet recognized as a coding gene. Mice harboring targeted deletion of either the ICR or *Percc1* recapitulated the human phenotype. Finally, we showed that *PERCC1* is critical for the function of EECs, elucidating the pathomechanism underlying IDIS.

Since the 2019 original description of eight affected individuals of seven unrelated families, only one additional affected individual was subsequently reported, presenting with diarrhea, failure to thrive, and parenteral nutrition (PN)-dependent intestinal failure (Maddirevula et al. 2020). Positional mapping and transcriptome sequencing identified a genomic deletion encompassing a regulatory element of *PERCC1* as the cause of disease. To date, however, no pathogenic single nucleotide variants in *PERCC1* have been reported in human disease.

We present herein a point mutation in *PERCC1* in association with a congenital enteropathy in two unrelated probands of Irish descent and discuss their molecular and phenotypic features.

## Methods

### Editorial policies and ethical considerations

The study was conducted in accordance with the tenets of the Declaration of Helsinki and approved by the Institutional Review Board (IRB) at the Sheba Medical Center and the Ethics Committee at Children’s Health Ireland at Crumlin. Written informed consent was obtained from the adult patient or minor patient’s guardian for publication of clinical and molecular data.

### Clinical data and sample collection

Clinical details were obtained from the patients' medical files. Whole blood samples were obtained from each participant into a 5 ml EDTA tube. DNA samples were extracted using automatic device MagNa Pure LC system. Concentrations and purity were measured on the Nanodrop ND-1000 instrument (Nanodrop Technologies, Wilmington, DE).

### Analysis for deletions

Initially, specific analysis for the two deletions encompassing the ICR was performed for each proband, as previously described (Oz-Levi et al. 2019). In brief, a multiplex PCR assay was performed for each of the two deletions. The boundaries of the long (ΔL) and short (ΔS) deletions, as previously determined using long PCR and Sanger sequencing, are: Chr16:1475369-1482326 and Chr16:1480850-1483951, respectively, yielding deletions of 6957 bp and 3101 bp, overlapping by 1476 bp. For the multiplex PCR, two PCRs were run: one long PCR yielding a band for a heterozygote subject, and one internal PCR designed to yield no band in case of a homozygote subject.

### Sanger sequencing and mutation analysis

PCR was carried out in a 25 µl reaction containing 50 ng of DNA, 10 pmol of each primer, ddH2O and Red Load Taq Master*5 (LAEOVA). After an initial denaturation of 5 min at 95 °C, 35 cycles were performed (94 °C for 30 s, 56–60 °C for 30 s, and 72 °C for 30 s), followed by a final extension of 10 min at 72 °C. The F-5′ GCGCCTGCACAAACACTCCA 3′, R-5′ GCCCAGTGGTCTGGGGGTCT 3′ primer set was used for amplifying the *PERCC1* non-coding exon 1 and the F-5′ ATCCGAGGGCAGCAGTCGAG 3′, R-5′ CCTTAGGGGTGGCCCTGCTG 3’ primer set was used for amplifying the coding sequence (exon 2). Sanger sequencing was performed at the Molecular Biology Service Laboratory, Hy-labs (Rehovot, Israel).

### Gastrin level measurement

Gastrin level were measured at two time points: First, from blood sample obtained from the patient after 8 h fast, and second, from blood samples obtained one hour after a small meal.

### Patient and public involvement

Both patients were selected for genetic testing of the ICR and *PERCC1* based on their chronic intestinal failure for which no cause had been identified. The rationale, risks and benefits of this targeted genetic testing was discussed with the patients and (for the minor patient) their parents. Following identification of the point mutation, patients and parents participated in discussions with the clinical team regarding best next steps from both a clinical and research perspective, thus leading to measurement of fasting and post-prandial serum gastrin levels, and the joint decision to publish these cases in a journal and present the findings for discussion at conferences with the wider medical community.

## Results

We encountered two unrelated Irish patients, recruited at the National Centre for Pediatric Gastroenterology, CHI-Crumlin, both suffering from a CODE of unknown etiology. Their clinical, laboratory and molecular characteristics are summarized in Table 1.

**Table 1:** Clinical, laboratory and molecular characteristics of two unrelated individuals harboring a disease-causing nonsense variant in *PERCC1*

Characteristics	Patient A	Patient B
Demographic and clinical data		
Sex	M	M
Current age (years)	12	19
Age at symptom onset	3 weeks	2 weeks
Presenting symptom(s)	Watery diarrhea, hypernatremic dehydration	Watery diarrhea, hypernatremic dehydration
Comorbidities	None	None
Parental consanguinity	N	Y
Ethnic origin	Irish (3/4), Sri Lankan (1/8), Malaysian (1/8)	Irish
Variant in *PERCC1*	Homozygous c.390C > G (Y130X)	Homozygous c.390C > G (Y130X)
Laboratory data		
Stool sample		
Sodium (mmol/L)	9	46
Chloride (mmol/L)	12	NA
Osmolarity (mOsm/L)	297	NA
Reducing substances	+++	++++
Elastase (µg/gr)	430	> 300
3-day fecal fat (gr/day)	298 (age 12 years)	18 (age 3 years)
Serum sample		
Zinc	Normal	Normal
Anti-enterocyte Ab	Negative	NA
Amino acid profile	Normal pattern	Normal pattern
Transferrin isoforms	Normal	Normal
Lactate	Normal	Normal
Pyruvate	Normal	Normal
Mitochondrial DNA sequencing	Normal	NA
Urine sample		
Organic acid profile	Normal	Normal
Catecholamines	Normal	NA
Purines and pyrimidines	Normal	NA
Histopathology		
EGD and ileocolonoscopy	Normal	Normal
PAS/CD10 stain (small bowel)	Normal	Normal
Chromogranin A / Synaptophysin (small bowel)	C/W control sample	NA
Additional investigations		
Liver biopsy (including EM and respiratory chain analysis)	Macro- and microvesicular steatosis (on PN)	Macro- and microvesicular steatosis (on PN)
Sweat Chloride (mmol/L)	13	12
Exome sequencing	Negative (trio)	Negative (proband only)

*EM* electron microscopy, *NA* not available, *EGD* esophagogastroduodenoscopy, *PAS* periodic acid-Schiff

### Clinical presentations

Patient A is a 12 year-old male, born to non-consanguineous parents of mixed Irish/Sri-Lankan/Malaysian descent (father Irish; mother 1/2 Irish, 1/4 Sri-Lankan, 1/4 Malaysian), with one healthy older sibling and two half-siblings. He was born weighing 3.18 kg following an uncomplicated term pregnancy and presented aged 3 weeks with watery diarrhea and hypernatremic dehydration. He underwent extensive investigations (Table 1), including negative trio-based exome sequencing (ES), and endoscopic biopsies which showed normal chromogranin A and synaptophysin staining and normal enteroendocrine cell (EEC) number (5–6 per crypt). Investigations did not support a metabolic or mitochondrial disorder. He is cognitively normal and growing appropriately on home parenteral nutrition (4 nights per week, 67 non-protein calories/kg, 56 ml/kg). He is hyperphagic and has a diverse diet but has 5–7 steatorrheal (298 g fat/day) stools per day. Serum gastrin levels were low and failed to respond to meal challenge [fasting and post-prandial gastrin 34 and < 33 ng/L, respectively (normal range 33–85 ng/L)].

Patient B is a 19 year-old male, born to consanguineous parents (first cousins) of Irish ethnicity. He presented at 2 weeks of age with watery diarrhea and hypernatremic dehydration following an uncomplicated pregnancy, birth weight 3.04 kg. Diarrhea resolved on cessation of oral feeds, and extensive investigations failed to identify a cause (Table 1). He is cognitively normal and has reached an adult height of 179.1 cm (75th centile). He is tapering his home PN regime, currently on PN 1 night/week (15 non-protein calories, 32 ml/kg) with a diverse oral diet, passing 3–5 loose stools/day. Serum gastrin levels were low and failed to respond to meal challenge [fasting and post-prandial gastrin 24 and 22 mU/L, respectively (normal range 23–113 mU/L)]. Single (proband only) exome sequencing was negative.

### Molecular analysis

Because of our previous work describing ICR deletions as the genetic cause of IDIS (Oz-Levi et al. 2019), we applied the candidate gene approach to seek pathogenic variants in this region. Both patients were found to be wildtype for the reported deletions. Since the reported deletions regulate *PERCC1*, we next performed Sanger sequencing of its coding region and intron boundaries (NM_001365310.2) and found a novel c.390C > G (p.Y130X) stop gain variant in *PERCC1*. The variant was found in homozygous state in both patients, and in heterozygous state in the available parents (both parents of Patient A, and the mother of Patient B).

### Frequency of the PERCC1 variant in public databases

The variant frequency in gnomAD v3, was 12 alleles among 152,240 individuals (0.00007). These included eleven heterozygous carriers from the European population and one from the Latino-American population. No homozygote individuals were identified.

## Discussion

The role of EEC dysfunction in diarrhea is an evolving field of particular relevance to patients with CODEs. Elucidation of the molecular basis of CODEs may not only provide insights on the roles of mutated genes in EEC differentiation and human gastrointestinal tract development, but may also shed light on more common disorders in which EECs are implicated, including obesity and diabetes (Gribble and Reimann 2019). Our findings provide the first description of a disease-associated point mutation in *PERCC1* in humans, complement our previous work implicating deletion of ICR in the etiology of CODEs and confirm that *PERCC1* is indeed a previously-unrecognized gene of clinical significance.

Importantly, while *PERCC1* has recently been cataloged in OMIM (MIM:618656), it remains unannotated in the hg19 built and is not covered by the most common exome enrichment kits currently in use. Thus, additional affected individuals undergoing exome sequencing will remain undiagnosed. It is noteworthy that both of the only two children with idiopathic congenital diarrhea attending the Irish National Intestinal Failure Program had *PERCC1* mutations. This suggests that *PERCC1* mutation may be a common cause of idiopathic CODEs worldwide, or that it is a founder mutation in the Irish population. While we were not able to determine this experimentally, future population-based genetic studies and reports of *PERCC1* testing in other CODE patients will shed some light on this.

In our original publication describing the first *PERCC1*-associated phenotype (Oz-Levi et al. 2019), we established a link between loss of *PERCC1* expression and EEC dysfunction. mRNA in situ hybridization of wildtype mice and examination of transgenic mice encoding a *PERCC1*-mCherry fusion protein indicated that *Percc1*-expressing cells were primarily EECs (including gastrin-secreting G cells in the stomach) in early fetal development. Further supporting the link between *PERCC1* and EEC function, RNAseq data revealed that *Percc1* knockout mice had significant reductions in *Gastrin*, *Somatostatin* and *Ghrelin* expression, as well as low serum gastrin levels. Consistent with these observations, an interesting clinical finding shared by both probands reported herein is that of low serum gastrin levels during fasting, which fail to respond to a meal challenge. Chromogranin A/synaptophysin staining in patient A did not demonstrate a reduction in intestinal EEC numbers. This is consistent with our previous effort, demonstrating increased immunoreactivity to Chromogranin A in biopsies obtained from the duodenal villi and stomach pyloric mucosae of two patients of the original cohort harboring the ΔL/ΔL deletion of ICR, when compared to healthy controls, as well as similar findings in the chr17^ΔICR/ΔICR^ mouse model (unpublished data). These findings suggest that EECs do form in early development in these patients, but it is their disrupted subsequent differentiation to EEC subtypes and their dysfunction that underlies the pathomechanism of this disorder. We can further speculate that the low gastrin levels seen in our current two patients may be an incidental marker of general EEC dysfunction, and that it is the intestinal EEC dysfunction which contributes to the clinical phenotype of diarrhea and intestinal failure. However, the mechanistic relevance of the clinical finding of low serum gastrin and its potential as a diagnostic clue in CODEs remain to be elucidated in further studies.

The c.390C > G variant observed in our two patients is a stop gain variant in the gene, where loss of function according to our original report is the mechanism of the disease. As *PERCC1* is expressed only in the earlier developmental stage, functional studies using patient-derived cells were not readily available. Recently, evolutionary bioinformatic analysis of *PERCC1* suggested that it is a member of the *YAP/TAZ/FAM181* family of homologous transcriptional regulators (Sanchez-Pulido et al. 2022). Future studies utilizing induced pluripotent stem cell (iPSC)-derived intestinal organoids and in vivo models would shed light on the yet-unanswered question of the precise role of *PERCC1* and its pathomechanistic underpinnings in human disease. Additionally, with the advent of enteroendocrine hormone analog treatments and better understanding of the mechanisms underlying diarrhea/malabsorption when these hormones are not produced, putative targeted therapeutic interventions can already be contemplated for patients with intestinal failure caused by EEC dysfunction (e.g. Glucagon-Like Peptide (GLP)-1 analogs, recently reported to improve malabsorptive diarrhea in patients with Mitchell-Riley syndrome (Nóbrega et al. 2021)).

While historically the clinical entity of autosomal recessive congenital diarrhea in families of Iraqi-Jewish descent was designated ‘Intractable Diarrhea of Infancy Syndrome’ (IDIS), in light of our findings and in line with current shifts in the nomenclature of genetic disorders, we suggest that this entity be termed ‘*PERCC1*-associated congenital enteropathy’. This term can apply to patients with either point mutations in *PERCC1* or genomic deletions regulating the gene.

To conclude, we report herein clinical and molecular findings in two unrelated patients harboring a shared homozygous variant in *PERCC1*, comprising the first description of a point mutation in this gene in association with CODE.

## Data Availability

The datasets generated during and/or analyzed during the current study are available from the corresponding author on reasonable request.
